# A Microbial Inoculum (*PLC-8*) Improves Composting of Spent Mushroom Substrate

**DOI:** 10.3390/microorganisms13112627

**Published:** 2025-11-19

**Authors:** Jiamin Yin, Hairu Yu, Sen Qi, Yufu Hu, Di Chen, Hongyan Zhao, Zongjun Cui

**Affiliations:** 1 College of Agriculture, Yanbian University, Yanji 133002, China; 2023050882@ybu.edu.cn (J.Y.); 2023050896@ybu.edu.cn (S.Q.); 2023010604@ybu.edu.cn (Y.H.); 13324332375@163.com (D.C.); 2 Characteristic Industry Development Center, Yanbian Korean Autonomous Prefecture, Yanji 133002, China; yuhairu@163.com; 3 College of Agriculture, China Agricultural University, Beijing 100193, China; acuizj@cau.edu.cn

**Keywords:** C/N ratio, lignocellulose-degrading bacteria, spent mushroom substrate, composting, microbial community

## Abstract

Composting is a useful way to reduce and recycle agricultural and forestry waste; however, low-temperature environments can inhibit the microbial processes involved in composting. Spent mushroom substrate has a high lignocellulose content, making it particularly difficult to decompose. There is a need to explore methods for effectively promoting microbial activity and enhancing composting efficiency under low-temperature conditions. This study explored the use of C/N ratio adjustments and a microbial inoculum (*PLC-8*; comprising Proteobacteria, Bacteroidetes, Actinobacteria, Firmicutes, Basidiomycota, Ascomycota, and Cryptomonadales) to improve spent mushroom substrate composting in a low-temperature environment. The temperature, lignocellulose content, pH, and gas emissions were measured during composting, and the microbial community structure was determined to explore associations between biotic and abiotic factors. Compost piles with *PLC-8* entered the high-temperature period in 25 days, which was 15 days earlier than the control pile. When the C/N ratio was adjusted to 30:1 and *PLC-8* was applied, the cellulose and hemicellulose degradation rates after 60 days were 88.04% and 71.95%, whereas the control group only exhibited degradation rates of 25.39% and 35.64%. Moreover, *PLC-8* significantly increased CH_4_ and CO_2_ emissions and reduced nitrous oxide emissions. Microbial community analysis showed that Proteobacteria and Ascomycota were the dominant phyla in the piles with *PLC-8*, and these phyla were responsible for lignocellulose decomposition and carbon metabolism.

## 1. Introduction

*Auricularia heimuer* is a type of black fungus that is widely cultivated in China, with the highest yield and fourth highest production of edible fungi in China [1,2]. However, the large-scale production of *A. heimuer* results in a huge amount spent mushroom substrate, which is an agricultural waste [2,3]. Spent mushroom substrate is rich in lignocellulose. Therefore, if not properly treated, spent mushroom substrate will decompose slowly and generate large amounts of greenhouse gases [4,5]. Conversely, spent mushroom substrate provides an important opportunity for nutrient recovery [4].

Low temperatures inhibit the activity and growth rate of microorganisms involved in lignocellulose degradation [6,7]. Therefore, agricultural and forestry waste products with high lignocellulose contents require specific treatments, which can include physical, chemical, and biological methods [8,9]. Biological methods, such as composting, utilize the degradation ability of microorganisms and are considered environmentally friendly and cost-effective [10,11]. However, in low-temperature environments, traditional composting processes are slow to start and cannot maintain high-temperature phases, resulting in low degradation efficiency and prolonged composting cycles [6]. To combat this, cold-tolerant or psychrophilic lignocellulose-degrading microbial inoculum can be added before composting [6,7]. Microbial inoculum can promote rapid temperature increases and effective degradation of lignocellulose during composting. This bioaugmentation strategy can shorten the composting cycle, improve the quality of compost products, and reduce greenhouse gas emissions [8]. For example, one study found that psychrophilic cellulose-degrading bacteria could effectively degrade corn straw at low temperatures [7]. In another study, co-application of biochar and microbial inoculum to compost significantly prolonged the thermophilic phase and enhanced the degradation of lignocellulose with final degradation rates for cellulose, hemicellulose, and lignin of 20.8–31.2%, 36.2–44.8%, and 34.2–43.5%, respectively [5]. These studies reveal a practical role of microbial inoculum in optimizing lignocellulose composting in low-temperature environments.

The C/N ratio of the substrate is a key factor during composting [9,10]. Microorganisms require balanced carbon and nitrogen sources for optimal metabolic activities [5,11]. A C/N ratio that is too high or too low will inhibit microbial activity, resulting in a decrease in decomposition efficiency [12]. Specifically, if the C/N ratio is too high, microorganisms will be nitrogen-limited and will not be able to effectively synthesize proteins, resulting in low lignocellulose decomposition efficiency. Moreover, microorganisms will preferentially utilize easily degradable carbon sources instead of decomposing complex carbon sources such as lignocellulose. Conversely, when the C/N is low, excess nitrogen volatilizes in the form of ammonia, causing nitrogen loss and pollution [13]. Therefore, optimizing the C/N ratio for composting can reduce nutrient loss and improve compost quality [14].

While composting is an effective method for reducing landfill and recycling nutrients, it inevitably results in greenhouse gas emissions and nitrogen loss [15]. CO_2_ is produced through microbial respiration; however, under anaerobic conditions, CH_4_ is produced, which is a potent greenhouse gas with a higher global warming potential [16]. The transformation of nitrogen is more complex; ammonification of organic nitrogen, nitrification of NH_4_^+^-N, and denitrification of nitrate nitrogen result in NH_3_ and N_2_O emissions [9,17]. The addition of microbial inoculum can promote aerobic decomposition, thereby reducing CH_4_ production [8]. Meanwhile, C/N ratio optimization can help to inhibit the generation of N_2_O and NH_3_, reducing nutrient loss and greenhouse gas emissions [9,13].

While various studies have confirmed that microbial inoculum and C/N ratio regulation can improve composting efficiency, most of them were conducted in medium- and high-temperature environments. Moreover, few studies have investigated the composting of spent mushroom substrate in low-temperature environments. We hypothesized that microbial inoculum addition and C/N ratio optimization can improve spent mushroom substrate composting under low temperatures. The objectives of this study were as follows: (1) to compare the impacts of different microbial inoculation amounts and C/N ratios on temperature dynamics, greenhouse gas emissions, nitrogen loss, cellulose degradation, and compost quality during the composting process; (2) to clarify the differences in compost microbial communities among various treatments; and (3) to elucidate the relationships between microbial communities and compost chemical properties, greenhouse gas emissions, and cellulose degradation. Our results provide technical guidance for improved utilization of edible fungi waste, thus promoting circular agriculture and sustainable development. However, there may be certain limitations in applying the findings of this study. For example, in different regions, under varying climatic conditions, and with different raw material characteristics, the applicability of the relevant technologies may require further validation and adjustment.

## 2. Materials and Methods

### 2.1. Composting Materials and Ratio

The experiment was conducted at Yanbian University, Yanji City (longitude 129.18° and latitude 43.10°), in a temperate semi-humid climate zone. The experimental materials were spent mushroom (*Auricularia heimuer*) substrate, fresh pig manure, and a microbial inoculum containing lignocellulose-degrading bacteria (*PLC-8*), which was obtained from leaf litter collected from a low-temperature environment. The inoculum contained a consortia of bacteria and fungi, including Proteobacteria, Bacteroidetes, Actinobacteria, Firmicutes, Basidiomycota, Ascomycota, and Cryptomonadales. Cultivation methods of *PLC-8*, analysis of microbial community diversity, and straw decomposition characteristics described by Yang et al. [18].

Five compost treatments were established (Table 1). First, spent mushroom substrate and pig manure were mixed together in a 3:1 ratio. Then, nitrogen (urea, N_2_O ≥ 46%) and *PLC-8* were added as follows: control (CK), no additional nitrogen or *PLC-8*; treatment A, C/N ratio adjusted to 25:1, no *PLC-8* added; treatment B, C/N ratio adjusted to 30:1, no *PLC-8* added; treatment AM, C/N ratio adjusted to 25:1, 10 mL of *PLC-8* added; treatment BM, C/N ratio adjusted to 30:1, 10 mL of *PLC-8* added. All compost treatments were mixed manually.

### 2.2. Composting Methods and Sampling

Five compost piles were constructed, each with a volume of 5 m^3^ and a height of 1.3 m. Fermentation began naturally. After the pile temperature reached 50 °C, the piles were manually turned over every 2 d. Then, after the temperature dropped to 35 °C, the piles were manually turned every 5 d. Piles were composted for 60 days, with day 0 representing the day that piles were constructed. Samples were taken every 2 days at depths of 30 cm, 80 cm, and 100 cm from the top of the pile. The diagonal five-point sampling method was used for each layer, and the samples were mixed evenly. Then, a 20 g sample was accurately weighed, dried at 105 °C, and the moisture content was calculated based on the weight loss. The remaining sample was air-dried in the dark for later use. Gas sampling was conducted at the initial stage of composting on days 0, 10, 40, and 60. The static chamber method was employed for greenhouse gas collection. Gas samples were collected using a 30 mL syringe at the 30th minute of both the aeration and intermittent periods during composting and stored in 12 mL headspace vials [19].

### 2.3. Measurement of Compost Pile Temperature

Compost temperature was measured at 10:00 a.m. and 12:00 noon every day during the composting process. Temperature sensors were inserted into the pile at depths of 30 cm, 80 cm, and 100 cm from the surface at 10:00 a.m. and at depths of 20 cm, 50 cm, and 70 cm at 12:00 noon. The ambient temperature was also measured at 10:00 a.m. and 12:00 noon every day.

### 2.4. Determination of Chemical Indicators

The pH was measured in a water:sample mixture at a 5:1 ratio using a pH meter (pH-100A, 100–2000 rpm, LICHEN, Shanghai, China) [20]. The organic matter (OM) content was determined using the potassium dichromate volumetric method combined with the dilution-heat approach. For total nitrogen (TN), samples were digested using the Kjeldahl method and nitrogen was measured using an AAIII continuous flow analyzer (AA3, AutoAnalyzer 3, Technicon, Windows/NT, SEAL, Harbin, China) [21]. For ammonium nitrogen (NH_4_^+^-N) and nitrate nitrogen (NO_3_^−^-N), samples were extracted using 2 mol/L CaCl_2_ and nitrogen pools were measured using an AAIII continuous flow analyzer [22]. The acid detergent fiber method was used to determine the cellulose (Cel), hemicellulose (Hcel), and lignin (Lig) contents [23]. Gas composition was determined using TDC (1000QPWRQ1, Dezhou, China) and gas chromatography [24].

### 2.5. Determination of Microbial Indicators

DNA was extraMicrobial diversity sequencing was then completed by Beijing Biomarker Technologies Corporation (Beijing, China). See Table S1 for specific methods.

### 2.6. Data Analysis

Data were organized using Excel 2016 (https://offlce.sujiee.cn/) (accessed on 3 August 2025). The microbial diversity Illumina PE300 (https://www.illumina.com) (accessed on 1 September 2025) analysis platform was used to analyze the diversity and correlations of bacteria and fungi. SPSS 21 (https://www.ibm.com/spss) (accessed on 6 August 2025) software was used to analyze compost physicochemical indicators. Structural equation modeling (SEM) was conducted using AMOS 26.0 (https://www.ibm.com) (accessed on 14 September 2025) to analyze the causal relationship between biotic and abiotic factors. The model fit was assessed using the χ^2^/df ratio (0.90) and RMSEA (<0.08). All charts were generated using OriginPro 2022 (https://www.originlab.com) (accessed on 6 September 2025), and *p* < 0.05 was considered significant.

## 3. Results

### 3.1. Temperature Change During Composting

**Figure 1:**
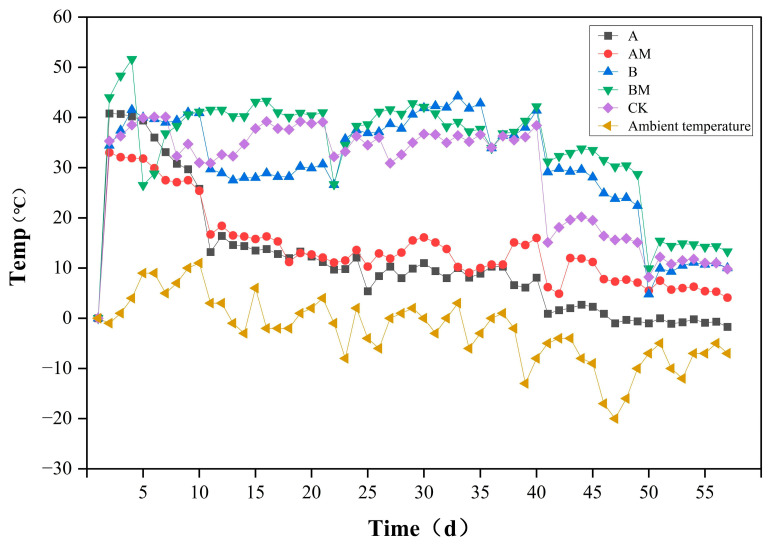
Compost temperature under different treatments during the composting process. A (C/N 25:1, without *PLC-8*), AM (C/N 25:1, with *PLC-8*), B (C/N 30:1, without *PLC-8*), BM (C/N 30:1, with *PLC-8*), and CK (control).

As shown in Figure 1, the temperature change curves of the compost piles indicate that the temperature changes in all treatments were closely related to the ambient temperature. The temperature of all treatments rose rapidly during the first 15 days and entered a high-temperature phase (>50 °C) on the 25th day. However, the AM and BM treatments, which received the *PLC-8*, reached the high temperature faster and remained at a high temperature for longer. Specifically, the high-temperature stage in the AM treatment lasted for about 10 days, which was significantly longer than that in the CK, A, and B treatments. This result indicates that, in low-temperature environments, inoculation with lignocellulose-degrading microbes effectively stimulates microbial activity and accelerates composting.

In the middle and late stages of composting (cooling stage; from day 15 to day 60), the AM and BM treatments had consistently higher temperatures than the corresponding uninoculated treatments (A and B) and the CK treatment. In particular, in the late stage of composting when ambient temperature fluctuated, the BM treatment exhibited higher temperatures. This result indicates that the *PLC-8* resulted in the continuous microbial degradation of organic matter. In contrast, the temperature of the CK treatment was the most passive, always closely following the ambient temperature. This result indicates low microbial activity, and hence low heat production, in the CK treatment. The temperature of the A and B treatments were between those of the CK and inoculation treatments. This result further confirms that although adjustments to the C/N ratio can impact compost temperature, microbial inoculation plays a decisive role in increasing and maintaining composting temperature in low-temperature environments.

In summary, the temperature data clearly show that microbial inoculation can effectively overcome the adverse effects of low-temperature environments, optimizing the composting temperature and achieving efficient and rapid composting of spent mushroom substrate.

### 3.2. Changes in Physicochemical Properties During Composting

During composting, there was an increase in the C/N ratio of the AM treatment, indicating that the carbon source was relatively abundant, while there was a decrease in the C/N ratio of the B and BM treatments, indicating the release of CO_2_ by carbon decomposition (Table 2). The AM treatment maintained a higher C/N ratio than the A treatment, which indicates that *PLC-8* plays a role in compost stabilization.

The CK treatment had an organic matter (OM) content of 319.74 g/kg. The OM content of treatment A was 357.13 g/kg at day 0, indicating that the C/N adjustments affected the OM content. After 60 days of composting, the OM content of treatment AM increased significantly to 514.77 g/kg, while the OM contents of treatments B and BM were 342.78 g/kg and 353.11 g/kg, respectively, with smaller increases. This result indicates that the addition of inoculum when the C/N ratio has been adjusted to 25:1 promotes microbial activity and the decomposition and accumulation of OM.

At day 0, the pH of the CK treatment was 7.7 and the pH of treatment A was 8.14. After 60 days of composting, the pH of each treatment slightly increased, with the pH of treatment AM being 8.18, treatment B being 7.98, and treatment BM being 7.92. The pH of all treatments was maintained between 7.9 and 8.2, indicating that the overall composting process was in a weakly alkaline environment. The changes in pH indicate microbial volatilization of ammonia. *PLC-8* had a small effect on pH. The pH of treatments A and AM were relatively high, likely because the higher C/N ratio promoted ammonification. The C/N of the CK treatment was 20.12. The C/N of treatment A was 21.13 at 0 day. After 60 days of composting, the C/N of treatment AM increased to 27.34, while the C/N of treatments B and BM decreased to 15.4 and 17.46, respectively.

The total nitrogen (TN) content of the CK treatment was 23.99 g/kg. The TN content of treatment A was 10.24 g/kg at day 0, possibly due to nitrogen dilution caused by C/N adjustments. After 60 days of composting, the TN of all treatments further decreased, with treatment AM at 12.18 g/kg, treatment B at 12.36 g/kg, and treatment BM at 10.61 g/kg. This decrease was likely due to nitrogen volatilization and denitrification, especially under low-temperature conditions, where microbial activity may not fully retain nitrogen. *PLC-8* had no significant effect on TN retention, but treatment BM had the lowest TN content, indicating that low C/N levels combined with *PLC-8* exacerbate nitrogen loss. The ammonium nitrogen (NH_4_^+^-N) content of the CK treatment was 6.06 mg/kg, and the nitrate nitrogen (NO_3_^−^-N) content was 140.96 mg/kg. Treatment A had a NH_4_^+^-N content of 24.11 mg/kg and a NO_3_^−^-N content of 7.38 mg/kg on day 0, indicating that the nitrogen form had changed after C/N adjustments. After 60 days of composting, the NH_4_^+^-N content in all treatments increased significantly: it was 24.7 mg/kg in treatment AM, 21.99 mg/kg in treatment B, and 21.93 mg/kg in treatment BM. This result indicates increased organic nitrogen mineralization. Meanwhile, the NO_3_^−^-N content decreased to 6.70–6.49 mg/kg in all treatments, indicating that nitrite nitrogen was further oxidized to nitrate nitrogen and participated in the denitrification process. This result also indicates active nitrogen cycling during composting. *PLC-8* promoted nitrogen conversion, but there were no significant differences between inoculated and un-inoculated treatments.

The contents of hemicellulose, cellulose and lignin in the CK treatment were 11.07%, 33.26% and 41.83%, respectively. Treatment A had different contents of these components at day 0 due to C/N adjustments. After 60 days of composting, the hemicellulose and cellulose contents of all treatments increased: to 52.49% and 46.28% for treatment AM, 61.66% and 52.32% for treatment B, and 84.04% and 71.95% for treatment BM, respectively. Meanwhile, the lignin content decreased: to 26.81% for treatment AM, 17.9% for treatment B, and 24.72% for treatment BM. This indicates that the composting process promoted the degradation of lignocellulose, especially the decomposition of lignin. *PLC-8* significantly enhanced the degradation of hemicellulose and cellulose, indicating that the enzymes excreted by the inoculum remained active at low temperatures. Treatments B and BM had the highest lignin degradation rates, with a higher rate in treatment BM.

### 3.3. Changes in Gas Emissions During Composting

**Figure 2:**
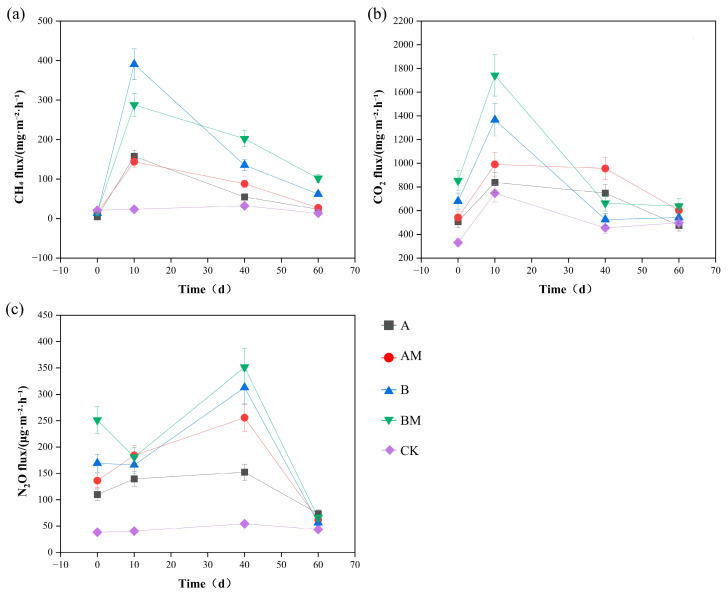
Greenhouse gas emissions during the composting process under different treatments: (**a**) CH_4_ flux, (**b**) CO_2_ flux, and (**c**) N_2_O flux. A (C/N 25:1, without *PLC-8*), AM (C/N 25:1, with *PLC-8*), B (C/N 30:1, without *PLC-8*), BM (C/N 30:1, with *PLC-8*), and CK (control). Data are presented as mean ± standard deviation (n = 3).

During composting, CH_4_ emissions generally increase and then decrease. If there is low aeration, resulting in insufficient or uneven distribution of oxygen, higher CH_4_ emissions occur. The CH_4_ emissions of each treatment during the composting process are shown in Figure 2a. Treatments A and B had higher CH_4_ emissions than treatment CK. *PLC-8* inhibited emissions during the pile heating stage but increased emissions during the high-temperature and maturation stages.

Microbial metabolism in compost can release CO_2_. Therefore, CO_2_ emissions directly reflect microbial OM decomposition. The CO_2_ emissions of each treatment during the composting process are shown in Figure 2b. Treatments A and B had higher CO_2_ emissions than treatment CK. *PLC-8* significantly increased CO_2_ emissions during the compost process. During the heating stage, the CO_2_ emissions of treatment B were higher than those of treatment A. The CO_2_ emissions of each treatment decreased from the high-temperature stage to the maturity stage. The overall microbial activity increased, which in turn increased CH_4_ and carbon dioxide emissions.

The N_2_O emission rate of each treatment during the composting process is shown in Figure 2c. There was no significant difference in emission rate between the early and late stages of composting (0 d and 60 d). The highest N_2_O emissions occurred during the 40 d high-temperature stage. The N_2_O emissions of treatments A, AM, B, and BM fluctuated greatly, indicating that thermophilic methane-oxidizing bacteria were undergoing nitrification and NH_3_ oxidation, resulting in more N_2_O production during the high-temperature stage.

There were no significant differences in emissions. In summary, both AM and BM had higher N_2_O emissions, indicating that *PLC-8* increases N_2_O emissions throughout composting. During the high-temperature phase, the CO_2_ emissions of the BM treatment were higher than those of the AM treatment, and the CO_2_ emissions of each treatment decreased sharply during the maturation phase. The emissions continued to decrease in the middle and late stages of the high-temperature phase until the end of composting. The N_2_O emission peaks for the AM and BM treatments were significantly different from the A and B treatments, indicating that the *PLC-8* caused a large consumption of oxygen.

The *PLC-8* significantly affected the greenhouse gas emission patterns under different C/N conditions. *PLC-8* promoted CO_2_ release, reflecting enhanced microbial activity and accelerated OM decomposition. *PLC-8* also inhibited CH_4_ emissions during the heating phase but increased them during the high-temperature and maturation phases. Moreover, *PLC-8* significantly increased the N_2_O emission peak, especially during the high-temperature phase, likely because *PLC-8* promoted nitrification and NH_3_ oxidation, resulting in oxygen competition. In summary, *PLC-8* and C/N regulation both affect compost aeration and microbial processes, thereby affecting carbon and nitrogen conversion pathways and gas emissions.

### 3.4. Changes in Microbial Community Composition During Composting

#### 3.4.1. Principal Component Analysis

**Figure 3:**
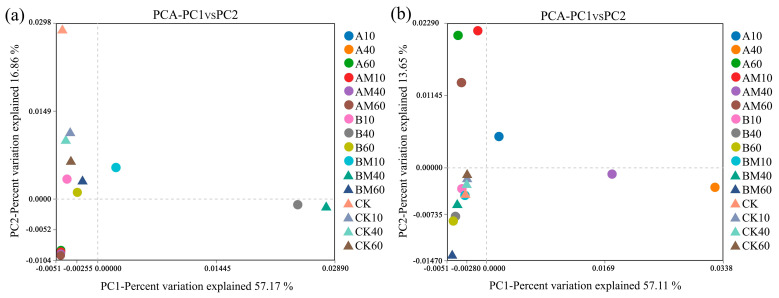
Principal component analysis (PCA) of microbial community structure during composting under different treatments: (**a**) bacterial community and (**b**) fungal community. A (C/N 25:1, without *PLC-8*), AM (C/N 25:1, with *PLC-8*), B (C/N 30:1, without *PLC-8*), BM (C/N 30:1, with *PLC-8*), and CK (control) at different composting times (10, 40, and 60 days). Values in parentheses on the axes indicate the percentage of variance explained by each principal component.

As shown in Figure 3, PCA indicated that the experimental treatments affected the microbial community in compost. Principal component 1 (PC1) explained 53.11% and 57.17% of the total variation in bacteria and fungi, respectively. For the bacterial community analysis, there was a clear separation of the CK treatments from the other treatments along PC1. For the fungal community analysis, there was a partial overlap between the CK treatment and the other treatments. These results indicate that bacteria and fungi had different responses.

#### 3.4.2. Microbial Community Phylum-Level Composition Analysis

**Figure 4:**
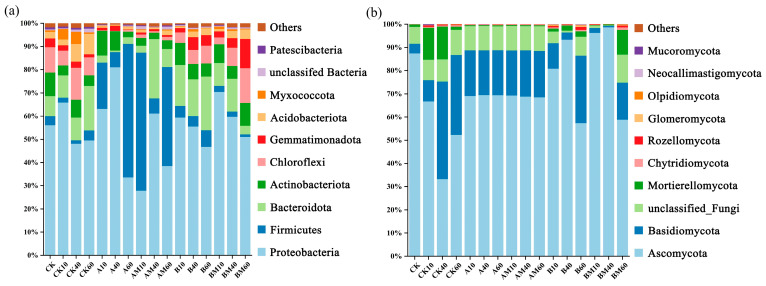
Relative abundance of microbial communities at the phylum level during composting under different treatments: (**a**) bacterial community and (**b**) fungal community. (A: C/N 25:1, without *PLC-8*; AM: C/N 25:1, with *PLC-8*; B: C/N 30:1, without *PLC-8*; BM: C/N 30:1, with *PLC-8*; CK: control) followed by the composting time in days (10, 40, 60). Only the top 10 most abundant phyla are shown, with the remaining taxa grouped into “Others”.

The bacterial community was analyzed at the phylum-level, and the results (Figure 4a) showed that Proteobacteria was the dominant bacterial phylum in all treatments. The *PLC-8* significantly increased the relative abundance of Proteobacteria. Given that *PLC-8* also increased CO_2_ emissions, this result indicates that Proteobacteria are key functional microorganisms driving the decomposition of AM and the production of CO_2_ during composting. In addition, nitrifying and denitrification bacteria in the phylum Proteobacteria are directly involved in nitrogen transformation and therefore could contribute to N_2_O emissions. Firmicutes, which is a typical anaerobic bacterial phylum, also had a high relative abundance in each treatment. These microorganisms are closely related to CH_4_ production under anaerobic conditions. The CH_4_ emission dynamics during composting followed a similar trend to the relative abundance of Firmicutes, suggesting that Firmicutes played an important role in CH_4_ production in this experiment.

The fungal community analysis at the phylum level (Figure 4b) showed that Ascomycota was the dominant phylum, and its relative abundance was significantly enriched by *PLC-8*. Ascomycota are important decomposers that can secrete a variety of extracellular enzymes to degrade complex organic matter such as cellulose and lignin, releasing CO_2_ in the process. The higher CO_2_ emissions in the treatments with *PLC-8* further indicates the central role of Ascomycota in carbon cycling and CO_2_ production during composting.

In summary, there was a close association between microbial phylum-level composition and greenhouse gas emissions. The structure of the microbial community directly determines its functional potential to participate in carbon and nitrogen cycles. The significant enrichment of Proteobacteria and Ascomycota in the treatments with *PLC-8* indicates a more efficient organic matter degradation system, which could explain the increase in CO_2_ emissions. Meanwhile, the changes in anaerobic bacteria such as Firmicutes were associated with the CH_4_ emission pattern. These results indicate that *PLC-8* optimized the microbial community structure, forming a more efficient organic matter degradation system that synergistically degrades lignocellulose. The enrichment of Proteobacteria and Ascomycota enhanced the aerobic degradation of cellulose, hemicellulose, and lignin, while Firmicutes was responsible for anaerobic fermentation. This synergy accelerates the conversion of lignocellulosic biomass, leading to increased CO_2_ and CH_4_ emissions. The results demonstrate the core role of microbial community structure in driving the conversion of lignocellulosic biomass, providing theoretical support for optimizing the composting process and reducing greenhouse gas emissions.

#### 3.4.3. Microbial Community Correlation Network Analysis at the Genus Level

**Figure 5:**
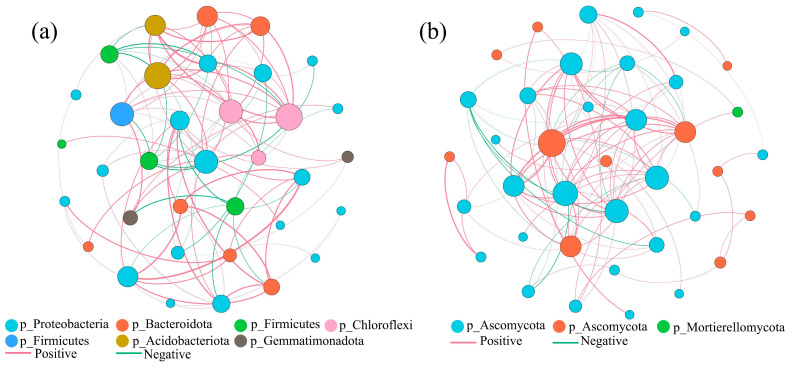
Microbial co-occurrence network analysis during composting under different treatments: (**a**) bacterial network and (**b**) fungal network. Node colors represent different microbial phyla (top 10 abundant phyla shown). Node size is proportional to the relative abundance of operational taxonomic units (OTUs). Edges represent significant correlations (Spearman’s |*r*| > 0.6, *p* < 0.05), with red and blue lines indicating positive and negative correlations, respectively.

The bacterial network is shown in Figure 5a. It covers multiple phyla such as Proteobacteria and Bacteroidota. The network has a large number of edges and a complex structure, which indicate a wide range of interactions within the bacterial community. The dominant phylum interactions were related to Proteobacteria and Acidobacteriota (yellow). The genera Pseudomonas and Sphingopyxis also had high connectivity. Nutrient competition and collaboration can result in positive and negative edges, regulating community function through carbon and nitrogen cycles. A large number of unclassified units participated in the network (such as unclassified_Vicinamibacteraceae), suggesting unknown interactions within the bacterial community and indicating environmentally specific functional groups.

The fungal network (Figure 5b) was dominated by Ascomycota, with a high proportion of nodes, and also included Mortierellomycota and other groups. The network was relatively focused, reflecting the group-specific interactions of the fungal community. Ascomycota-dominated unclassified_Agaricomycetes and Alternaria were at the core of the network, with dense positive correlation edges, indicating that Ascomycota interactions enhance organic matter decomposition; for example, Mortierella had a negative correlation with Ascomycota, affecting community structure through nitrogen resources competition.

#### 3.4.4. Microbial Community Association Heatmap

**Figure 6:**
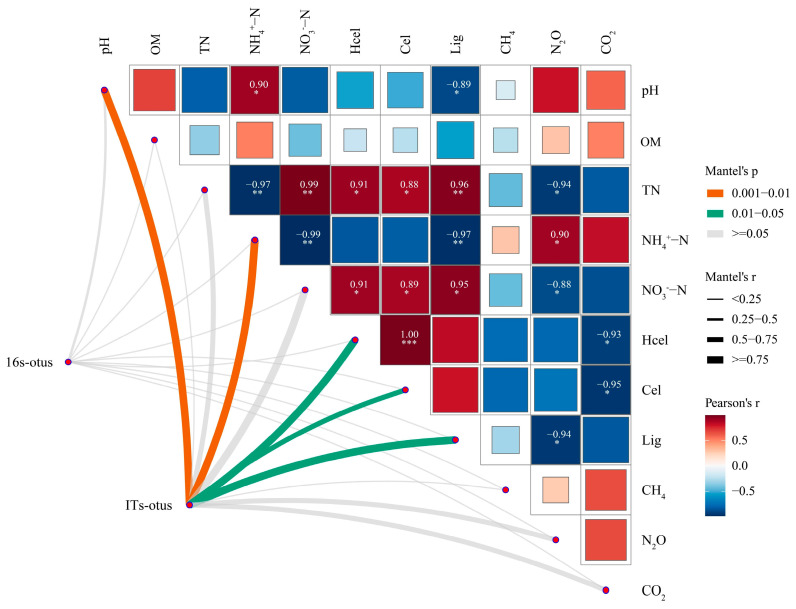
Correlation heat map. Rows represent different environmental factors, and columns represent bacterial community structure (16S-OTUs) and fungal community structure (ITS-OTUs). The color scale on the right represents the Pearson correlation coefficient (Pearson’s r), with blue indicating a positive association and red indicating a negative association; the deeper the color, the stronger the association. The figure also shows the Mantel test statistic (Mantel’s r) and significance range (Mantel’s p), which are used to assess the overall association between environmental factors and microbial community structure. OM (organic matter), TN (total nitrogen), NH_4_^+^-N (ammonium nitrogen), NO_3_^−^-N (nitrate nitrogen), Hcel (hemicellulose), Cel (cellulose), Lig (lignin). Different symbols *, **, and *** indicate significant differences at the *p* < 0.05, *p* < 0.01, and *p* < 0.001 levels, respectively.

The bacterial community exhibited significant associations with lignocellulosic components (Figure 6). Hemicellulose showed a positive association with the bacterial community, indicating that with changes in hemicellulose content, the bacterial community structure undergoes corresponding adjustments. This is likely because *PLC-8* promotes the degradation and utilization of hemicellulose by bacteria, thereby affecting the community composition. In addition, the bacterial community also has a strong association with nutrient factors such as OM and TN. OM was significantly positively correlated with the bacterial community, indicating that bacteria play an important role in the accumulation and transformation of OM during composting. The fungal community was associated with CH_4_ and N_2_O emissions. The CH_4_ emission rate was positively correlated with the fungal community composition, indicating that the metabolic activity of fungi promoted CH_4_ production during composting, which was consistent with the increased CH_4_ emissions in treatments with *PLC-8*. N_2_O was negatively correlated with the fungal community composition, indicating that there was a microbial mechanism driving the reduced N_2_O emissions in the treatments with *PLC-8*. Fungal community composition was negatively correlated with NH_4_^+^-N and positively correlated with NO_3_^−^-N, suggesting that fungi participated in the transformation of NH_4_^+^-N to nitrate nitrogen during composting, thus playing an important role in the nitrogen cycle. There was a significant positive correlation between TN and OM, indicating that there was a synergistic effect between nitrogen and OM transformation during composting, which may be related to microbial metabolic processes. Lignin was positively correlated with CO_2_, reflecting the carbon mineralization and gas release processes accompanying lignin degradation and explaining the intrinsic link between *PLC-8*-enhanced lignocellulose decomposition and increased CO_2_ emissions.

#### 3.4.5. Structural Equation Model (SEM)

**Figure 7:**
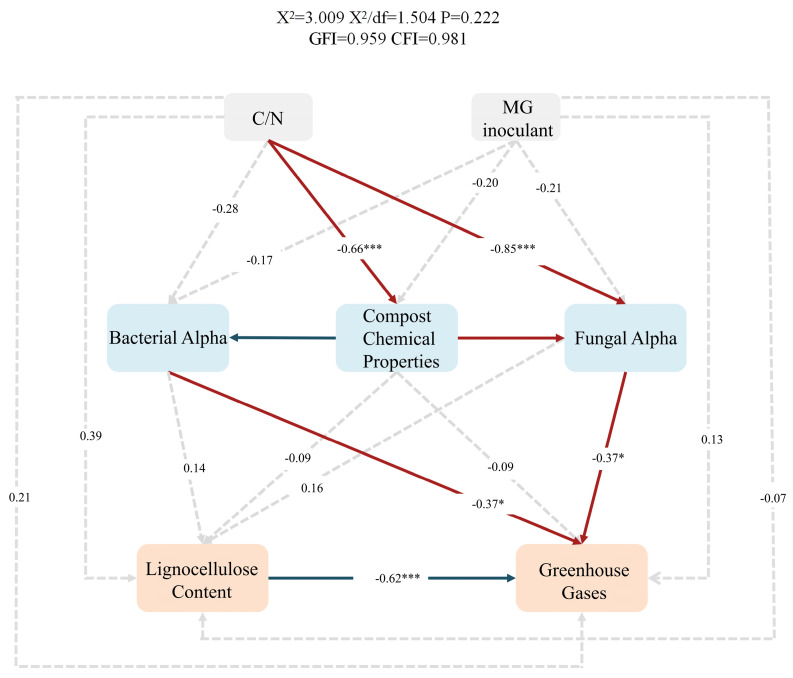
Structural equation modeling (SEM) results. The direct and indirect effects of the C/N ratio and *PLC-8* on lignocellulose content and greenhouse gas emissions during composting are shown. Blue and red indicate negative and positive correlations, respectively (* *p* < 0.05, *** *p* < 0.01); black dashed lines indicate no significant correlation.

The C/N ratio had a strong positive effect on composting properties (path coefficient 0.66, *p* < 0.001) (Figure 7), which is consistent with the notion that the C/N ratio is a key physicochemical factor in the composting process, directly regulating the decomposition of OM, the transformation of nutrients, and other chemical processes. The C/N ratio shapes the composting environment by altering cycling dynamics and the nutrient balance. The bacterial α diversity had a weak negative effect on greenhouse gases (−0.37, *p* < 0.05), suggesting that the bacterial community structure affects OM decomposition and thus greenhouse gas production. A high bacterial diversity may promote a more balanced decomposition of OM and reduce extreme decomposition pathways. Bacterial communities have functional redundancy and are adaptable, and their regulation effect on greenhouse gases is weaker than that of physicochemical factors. Lignocellulose content had a significant negative effect on greenhouse gases (−0.62, *p* < 0.001). This is likely because the recalcitrant nature of lignocellulose can inhibit rapid microbial decomposition and reduce the amount of gas produced by OM decomposition. In summary, the bacterial community composition is a key determinant of greenhouse gas emissions during composting and the final compost quality.

## 4. Discussion

### 4.1. Changes in Physicochemical Properties During Composting

The changes in physicochemical properties during composting are closely related to microbial communities and nitrogen transformation. The results of this study indicate that the application of the microbial inoculant *PLC-8* can significantly affect the key physicochemical properties during the composting process and promote nitrogen transformation, which is closely related to dynamic changes in microbial communities in the composting system. The application of *PLC-8* accelerated the time it took compost piles to enter the high-temperature phase, with piles reaching a peak temperature of 46.8 °C on day 40 [25]. The high-temperature phase lasted until day 43, indicating a significant shortening of the composting cycle. This result is likely due to the role of lignocellulose-degrading bacteria, which can accelerate substrate conversion and enhance microbial metabolic activity [5].

The extended thermophilic phase is crucial for compost safety, effectively killing pathogens and parasite eggs. During composting, the pH values of each treatment group fluctuated within the range of 7.7–8.3, providing a slightly alkaline environment for microbial growth. The pH values of treatments A and AM decreased first and then increased, which is related to the generation of organic acids and subsequent ammonification [11]. However, the pH value of the control (CK) treatment continued to rise, indicating its lower microbial activity and single metabolic pathway. *PLC-8* significantly improved the degradation efficiency of organic matter. The organic matter content in treatment A was the highest on the 10th day, which is related to the preferential decomposition of cellulose by the microorganisms in *PLC-8* under a high C/N ratio [5].

During the late stage of composting (days 40–60), the CK treatment exhibited the highest residual organic matter content at 7.99 g/kg, and there was no significant difference between the CK and BM treatments. This indicates that the combined use of a low C/N ratio and *PLC-8* can promote humification during the late stage [26]. An appropriate C/N ratio can balance the carbon and nitrogen requirements of microorganisms, thereby avoiding carbon excess or nitrogen limitation. The total nitrogen content generally decreased, which is likely related to ammonia volatilization [27]. However, the AM treatment had the highest total nitrogen retention at 33.38 g/kg, indicating that the C/N ratio of this treatment was more conducive to nitrogen fixation [26]. The treatments with *PLC-8* had significantly higher ammonium nitrogen content than the CK treatment on day 60 (the AM treatment reached 24.70 mg/kg), suggesting that the inoculum reduced nitrogen loss by enhancing ammonia assimilation [28].

The significant reduction in nitrate nitrogen further confirms the inhibition of the denitrification process by the microorganisms in *PLC-8* or the preferential utilization of nitrate nitrogen by microorganisms. This is closely related to the regulation of the composting microenvironment. *PLC-8* accelerates the degradation of lignocellulose, promoting the release of these nutrients [29]. The stability of available potassium and available phosphorus indicates that the inoculant can alleviate the leaching of available nutrients. Especially under low C/N ratio conditions, the enhanced microbial metabolic activity helps to refine nutrient forms [30]. The application of microbial inoculants, combined with appropriate C/N ratio adjustment, can not only accelerate the composting process but also improve nutrient retention and humification degree, ensuring efficient utilization and minimization of nutrient loss during composting [31].The treatments with *PLC-8* entered the high-temperature phase more quickly, which was attributed to the accelerated substrate transformation by lignocellulose-degrading bacteria [25].

Overall, composting is a complex process in which the changes in microbial communities and physicochemical properties are closely related [32]. By regulating the C/N ratio and adding *PLC-8* during composting, the degradation and humification of organic matter can be effectively promoted, and nutrient loss can be reduced, thereby improving the quality of compost.

### 4.2. Changes in Lignocellulose During Composting

The degradation of hemicellulose showed significant stage-specific characteristics. In the initial stage of composting (0–10 days), the hemicellulose content of the AM treatment group was the lowest (14.84%) and was significantly lower than that of the CK group (>30); the low hemicellulose content likely inhibited microbial activity [33]. By the intermediate stage of composting (40 days), the hemicellulose content in all treatments tended to converge (approximately 16.75%), reflecting that the microbial community reached metabolic equilibrium in the mesophilic phase. Notably, the BM treatment had the lowest hemicellulose content at the end of composting (60 days; 11.96%), suggesting that the combined use of a high C/N ratio and inoculum was more advantageous in long-term degradation. The inoculum compensated for the initial excess of carbon due to the high C/N ratio and simultaneously promoted the synergistic decomposition of the lignin-hemicellulose complex [6]. The degradation of cellulose exhibited a two-stage pattern, a rapid decomposition phase (0–40 days) and a stable phase (40–60 days). During the rapid decomposition phase, the cellulose content in the BM treatment decreased from 39.40% to 19.45%, a reduction of 50.6%, which was significantly higher than that in other treatments, forming a positive feedback loop.

Lignin, due to its complex aromatic structure and hydrophobicity, is the most recalcitrant component in composting. During short-term composting (10–40 days), the lignin degradation rate in the AM treatment was significantly higher than that in other treatments (*p* < 0.05), which was likely related to the low C/N ratio promoting the secretion of laccase and manganese peroxidase by white-rot fungi. However, in long-term composting (60 days), the degradation efficiency of the BM treatment, which had a high C/N ratio, gradually became prominent, indicating that the microorganisms in the inoculant continuously secreted lignin peroxidase to cleave carbon, while the high C/N ratio reduced the competitive inhibition of nitrogen on lignin-degrading enzymes [19]. These findings challenge the traditional view that a low C/N ratio is more conducive to lignin degradation, suggesting that the C/N ratio needs to be dynamically regulated in long-term composting to balance enzyme activity and substrate availability.

### 4.3. Changes in Gas Emissions During Composting

In the composting process, gas emission characteristics are key indicators reflecting microbial activity and organic matter transformation. Regarding CH_4_ emissions, this study showed that different C/N ratio treatments (A and B) could increase CH_4_ emissions from the compost, and the overall trend was first increasing and then decreasing, which is consistent with the degradation pattern of organic matter during composting. An insufficient ventilation rate or uneven oxygen distribution can significantly increase CH_4_ emissions [15], indicating that oxygen supply has an important impact on the activity of methanogens. The addition of cellulose-decomposing bacteria significantly inhibited CH_4_ emissions during the heating phase but showed an increasing trend during the high-temperature and maturation phases, which is related to the activity of the bacteria at different temperature stages and their role in promoting the decomposition of organic matter.

CO_2_ is a product of microbial metabolism, and its release directly reflects the microbial activity and organic matter decomposition rate in the compost. Both treatments A and B increased CO_2_ emissions, and the addition of cellulose-decomposing bacteria significantly increased CO_2_ emissions throughout the fermentation process [34], indicating that these treatments enhanced overall microbial activity and accelerated organic matter decomposition. The CO_2_ emissions from treatment B were higher than those from treatment A during the heating phase. However, the CO_2_ emissions of each treatment showed a decreasing trend from the high-temperature phase to the maturation phase, which was related to the change in compost temperature and the decrease in organic matter availability.

N_2_O emissions were mainly concentrated in the high-temperature phase of composting at day 40, and the emissions of the A, AM, B, and BM treatments fluctuated greatly, which was the result of nitrification and ammonia oxidation by thermophilic methane-oxidizing bacteria.

The AM and BM treatments increased N_2_O emissions, and the addition of cellulose-decomposing bacteria significantly increased the N_2_O emissions throughout the fermentation process. The N_2_O emissions of the BM treatment were higher than those of the AM treatment during the high-temperature phase but decreased sharply during the maturation phase. The N_2_O emission peak of the treatment with added cellulose-decomposing bacteria was different from that of other treatments, which is speculated to be due to the large consumption of oxygen in the pile by the bacteria, affecting the nitrogen conversion process of related microorganisms [35].

### 4.4. Changes in Microbial Community During Composting

PCA (Figure 3) showed that the β-diversity of bacterial and fungal communities was significantly affected by the C/N ratio and *PLC-8*. For the bacterial community analysis, PC1 (53.11%) and PC2 (13.65%) explained 66.76% of the variation, and the main differentiation appeared between different treatments, reflecting the regulatory effect of the C/N ratio on carbon source allocation [36]. For the fungal community analysis, PC1 (57.17%) and PC2 (16.86%) explained 73.03% of the variation, indicating that fungi were more sensitive to the nitrogen enhancer (*PLC-8*). The fungal community in the AM treatment deviated significantly from that in the CK treatment, which was related to the inoculant promoting lignin degradation and releasing nitrogen-containing precursor substances [37]. The dominant bacterial phyla were Proteobacteria, Firmicutes, and Bacteroidota (Figure 4), and the abundance of these groups increased significantly during the high-temperature period, which was closely related to their heat resistance and fiber degradation ability [38].

The addition of *PLC-8* increased the relative abundance of Actinobacteriota in the late stage (60 days), which likely contributed to the stabilization of compost products due to its involvement in humus synthesis and antibiotic secretion [39]. The dominant fungal phyla were Ascomycota and Basidiomycota. Correlation network analysis (Figure 5) showed that the core bacterial genera (e.g., *Brevundimonas* and *Sphingomonas*) mainly originated from Proteobacteria, and their interaction relationships (Spearman *p* > 0.1) reflected the synergy of carbon metabolic pathways (e.g., glycolysis and the TCA cycle). These genera accelerate cellulose hydrolysis by secreting extracellular enzymes and provide intermediate metabolites for other groups (e.g., Actinobacteria). The negative correlation (*p* < −0.1) between the core fungal genera (e.g., *Pseudeurotium*) and bacteria resulted from resource competition, but also implied a complementary effect of cross-domain interactions on lignocellulose complex degradation [40]. The stability of core microbiota during long-term composting and its beneficial effects on soil health.

## 5. Conclusions

This study provides evidence of the ability of a microbial inoculum (*PLC-8*; comprising Proteobacteria, Bacteroidetes, Actinobacteria, Firmicutes, Basidiomycota, Ascomycota, and Cryptomonadales) to accelerate spent mushroom substrate composting under low-temperature conditions. *PLC-8* significantly improved the degradation of cellulose and hemicellulose, driving lignocellulosic biomass conversion by the microbial community, optimizing the microbial community structure dominated by Proteobacteria and Ascomycota, and achieving environmental benefits by influencing gas emissions. Thus, this study provides strong technical support and a theoretical basis for the sustainable utilization of agroforestry wastes in cold regions.

## Figures and Tables

**Table 1:** Treatment description. A (C/N 25:1, without *PLC-8*), AM (C/N 25:1, with *PLC-8*), B (C/N 30:1, without *PLC-8*), BM (C/N 30:1, with *PLC-8*), and CK (control).

Treatment	C/N	*PLC-8*
A	25:1	Not added
AM	25:1	Added
B	30:1	Not added
BM	30:1	Added
CK	Untreated	

**Table 2:** Chemical properties of different compost treatments. A (C/N 25:1, without *PLC-8*), AM (C/N 25:1, with *PLC-8*), B (C/N 30:1, without *PLC-8*), BM (C/N 30:1, with *PLC-8*), CK (control), OM (organic matter), TN (total nitrogen), NH_4_^+^-N (ammonium nitrogen), NO_3_^−^-N (nitrate nitrogen), Hcel (hemicellulose), Cel (cellulose), Lig (lignin).

Time	Treatment	C/N	pH	OM (g/kg)	TN (g/kg)	NH_4_^+^-N (mg/kg)	NO_3_^−^-N (mg/kg)	Hcel (%)	Cel (%)	Lig (%)
0 d	CK	20.1	7.7 ± 0.4 c	319.9 ± 0.2 e	24.0 ± 2.7 a	6.1 ± 0.8 c	141.0 ± 0.3 a	11.1 ± 0.5 a	33.3 ± 0.2 a	41.8 ± 0.8 a
60 d	CK	18.5	8.0 ± 0.1 b	335.3 ± 0.5 e	9.1 ± 0.9 d	18.2 ± 0.5 b	11.5 ± 0.5 b	8.3 ± 0.2 b	22.5 ± 0.9 b	27.8 ± 0.6 d
	A	21.1	8.1 ± 0.7 a	357.1 ± 0.2 b	10.2 ± 1.2 c	24.1 ± 0.7 a	7.8 ± 0.2 c	5.4 ± 0.1 c	21.1 ± 2.1 b	30.8 ± 3.4 c
	AM	27.3	8.2 ± 0.2 ab	514.5 ± 0.5 a	12.2 ± 1.3 b	24.7 ± 0.6 a	6.7 ± 0.1 d	5.3 ± 0.2 c	17.8 ± 0.2 bc	30.9 ± 0.2 c
	B	15.4	8.0 ± 0.1 b	342.6 ± 0.6 d	12.4 ± 4.5 b	22.0 ± 0.8 a	6.7 ± 0.3 d	4.2 ± 0.7 d	15.8 ± 0.2 c	33.9 ± 0.2 b
	BM	17.5	7.9 ± 0.1 b	353.5 ± 0.9 c	10.6 ± 1.6 c	21.9 ± 1.1 a	6.5 ± 0.6 d	1.8 ± 0.6 e	9.3 ± 0.9 d	31.4 ± 0.3 bc

Different lowercase letters within the same column indicate significant differences among treatments (*p* < 0.05).

## Data Availability

The original data presented in the study are openly available in Zenodo at https://zenodo.org/records/17422329 (accessed on 23 October 2025).

## Supplementary Materials

The following supporting information can be downloaded at: https://www.mdpi.com/article/10.3390/microorganisms13112627/s1, Table S1: PCR amplification procedure for soil bacteria and fungi; Table S2: Amplification primer sequences for soil bacteria and fungi.
