# Supplementary material for: A Microbial Inoculum (PLC-8) Improves Composting of Spent Mushroom Substrate
Source: Microorganisms. 2025 Nov 19;13(11):2627. doi: 10.3390/microorganisms13112627 (PMC12654449; doi:10.3390/microorganisms13112627)
Supplement: Supplementary file 1 [file microorganisms-13-02627-s001.zip › microorganisms-3973674-supplementary.pdf]

Table S1.PCR amplification procedure for soil bacteria and fungi

Reaction Step	Temperature and Time
①Initial Denaturation	95℃ 5 min
②Denaturation	95℃ 30 s
③Annealing	50℃ 30 s
④Extension	72℃ 40 s
⑤Final Extension	72℃ 7 min
⑥Hold	4℃ +∞

Table S2. Amplification primer sequences for soil bacteria and fungi

Amplification region	Primer sequence
16S rDNA V3–V4 (338–806)	ACTCCTACGGGAGGCAGCA<br>GGACTACHVGGGTWTCTAAT
ITS1	CTTGGTCATTTAGAGGAAGTAA<br>GCTGCGTTCTTCATCGATGC
